# Metabolic profiling reveals metabolic features of consolidation therapy in pediatric acute lymphoblastic leukemia

**DOI:** 10.1186/s40170-023-00302-6

**Published:** 2023-01-23

**Authors:** Jinqiu Fu, Aijun Zhang, Qinqin Liu, Dong Li, Xiaoming Wang, Libo Si

**Affiliations:** 1 Department of Pediatrics, Qilu Hospital of Shandong University, Jinan, People’s Republic of China; 2 Department of Thoracic Surgery, Qilu Hospital of Shandong University, Jinan, People’s Republic of China

**Keywords:** Metabolomics, Acute lymphoblastic leukemia, Consolidation therapy

## Abstract

Acute lymphoblastic leukemia (ALL) and its treatment continue to pose substantial risks. To understand ALL more deeply, the metabolome in fasting plasma of 27 ALL patients before and after high-dose methotrexate therapies (consolidation therapy) including methotrexate and 6-mercaptopurine (6-MP) was investigated. Plasma metabolites were analyzed using liquid chromatography–tandem mass spectrometry (LC–MS). Orthogonal projections to latent structures discriminant analysis and significance analysis of microarrays were used to evaluate the metabolic changes. Pathway enrichment and co-expression network analyses were performed to identify clusters of molecules, and 2826 metabolites were identified. Among them, 38 metabolites were identified by univariate analysis, and 7 metabolites that were altered by conditioning therapy were identified by multivariate analysis. The Kyoto Encyclopedia of Genes and Genomes (KEGG) database was used for pathway enrichment analysis. Among the enriched KEGG pathways, the 3 significantly altered metabolic pathways were pyrimidine metabolism; phenylalanine, tyrosine, and tryptophan biosynthesis; and phenylalanine metabolism. In addition, L-phenylalanine was significantly correlated with blood urea nitrogen (BUN), and palmitoylcarnitine was correlated with aspartate aminotransferase (AST). In summary, consolidation therapy significantly affected pyrimidine- and phenylalanine-associated metabolic pathways in pediatric ALL patients. These findings may provide an insight into the role of metabolic profiling in consolidation treatment and as a potential for pediatric ALL patients.

**Supplementary Information:**

The online version contains supplementary material available at 10.1186/s40170-023-00302-6.

## Introduction

Acute lymphoblastic leukemia (ALL) is a common malignant tumor in children globally, and its incidence is increasing annually [1]. Although advancements in treatment have led to a cure rate of up to 80% [2], patients remain at risk of adverse events, leading to morbidity or death from ALL and its treatments. Reducing these risks may improve outcomes [3]. Studies have addressed the mitigation of adverse events resulting from complex ALL therapies [4, 5] in an attempt to reduce mortality from adverse events.

There are three major phases of ALL therapy: induction, consolidation, and maintenance. The purpose of consolidation is to further intensify the elimination of leukemia cells after the eradication of leukemia cells by induction therapy. The main categories of drugs given during consolidation include 6-mercaptopurine (6-MP) and methotrexate (MTX), which are called high-dose methotrexate (HDMTX) treatment/therapy [6]. However, both drugs have toxicity. MTX is a versatile antineoplastic and immunosuppressive agent that exerts anticancer effects by inhibiting folate metabolism and nucleotide biosynthesis [7]. MTX is associated with severe toxicity, for example, acute kidney injury [8] and/or oral mucositis [9], which can prolong chemotherapy and increase the risk of ALL relapse. In the study, the MTX dosage in consolidation therapy, which depends on the risk grouping, ranges from 3 to 5 g/m^2^ according to ALL protocol [10]. 6-MP cytotoxicity causes hematotoxicity, hepatotoxicity, and nephrotoxicity mediated by thioguanine nucleotide metabolites [11]. However, little is known about their effects on host metabolism.

Metabolomics, which studies the metabolic profiles of diseases, as well as tissues, cells, urine, and blood, is a powerful tool for discovering diagnostic metabolites and biomarkers and for examining adverse reactions to anticancer drugs [12]. Saito et al. [13] analyzed plasma metabolites and complex lipids from 50 ALL patients during initial and post-induction therapy, using high-resolution tandem mass spectrometry (MS/MS) and differential mobility MS/MS. They identified more than 1200 metabolites and complex lipids on global metabolomics and lipidomics platforms, and the results suggested that docosahexaenoic acid–containing (22:6) triacylglycerols were decreased in the post-induction therapy. Yang et al. [14] investigated the plasma metabolites in 19 adult B-cell ALL patients along with 19 healthy donors using nuclear magnetic resonance–based metabolomics, and they identified a total of 35 differential metabolites that were enriched in glycolysis, gluconeogenesis, amino acid metabolism, fatty acid metabolism, and choline phospholipid metabolism. In addition, the optimal combination of choline, tyrosine, and unsaturated lipids was potentially used for the prognosis and prognostic prediction of adult B-cell ALL. Brown et al. [15] used gas chromatography–MS and liquid chromatography (LC)–MS for global metabolic profiling of cerebrospinal fluid samples from pediatric ALL patients, and it was found that glutamatergic pathways or oxidative stress might be responsible for ALL-associated fatigue. To gain greater insights into the metabolic changes that occur during HDMTX therapy, we investigated plasma metabolites using reversed-phase LC–MS-based metabolomics and identified 7 differential metabolites following HDMTX treatment. The correlations between major metabolites and clinical markers of blood, liver, and kidney function and MTX concentration were addressed. L-Phenylalanine was significantly correlated with BUN, and palmitoylcarnitine was significantly correlated with AST. Finally, pathway analysis showed that HDMTX treatment predominantly affected pyrimidine metabolism; phenylalanine, tyrosine, and tryptophan biosynthesis; and phenylalanine metabolism, which might provide insight into the role of metabolic profiling in consolidation treatment and as a potential for pediatric ALL patients.

## Materials and methods

### Blood collection and sample preparation

A total of 27 patients (all the patients treated with the same protocol from January 2021 to May 2021) were enrolled and treated using the CCCG-ALL2020 protocol (a modification of the CCCG-ALL2015 protocol) [16] at Shandong University Qilu Hospital, China. The study was approved by the Ethics Committee of Shandong University Qilu Hospital. Written informed consent was obtained from each participant’s parent or legal guardian, prior to enrollment and treatment.

Fifty-four fasting blood samples were obtained before HDMTX therapy (day 1, used as the control samples) and after HDMTX therapy (day 3). Metabolite levels reflect the prandial state, and eating may affect metabolic levels and activation of metabolic pathways in the body [17]. The use of fasting samples therefore strengthened the protocol. Samples were collected in EDTA tubes. For sample processing, blood was centrifuged at 1700 ×*g* for 7 min at room temperature. The plasma was isolated, aliquoted, and frozen at −80 °C.

### Metabolite extraction and quality control (QC) preparation

Plasma samples were thawed and vortexed for 30 s. Each sample (200 μL) was extracted using MeOH to acetonitrile (1:1, v/v), vortexed for 30 s, and then sonicated for 10 min. Proteins were precipitated after the samples were incubated at −20 °C for 1 h, followed by centrifugation at 20,000 ×*g* for 15 min at 4 °C. The supernatant was removed and dried using a vacuum concentrator. The extracts were dissolved in acetonitrile to H_2_O (1:1, v/v), then vortexed for 30 s, and sonicated for 10 min. The extracts were then centrifuged for 15 min at 20,000 rpm and 4 °C, and the insoluble debris was removed. The supernatants were transferred to HPLC vials and stored at −80 °C. Ten microliters of each sample was pooled to prepare the QC samples. The same extraction procedure was performed for QC sample preparation as described previously [15].

### LC–MS/MS and total ion current chromatography

Samples were separated on an amide column, using mobile phase A which consists of water mixed with 25 mM ammonium acetate and 25 mM ammonium hydroxide and mobile phase B acetonitrile. The injection volume was 4 μL, and the flow rate was 0.4 mL/min. The generic HPLC gradient is detailed in Supplementary Table 1. Mass spectrometry analysis was conducted using Q-Exactive MS/MS in both ESI-positive and ESI-negative ion modes. The probe-tuning parameters were as follows: auxiliary gas heater temperature, 400 °C; sheath gas, 40; auxiliary gas, 13; spray voltage, 3.5 kV for positive and negative modes; capillary temperature, 350 °C; and S-lens, 55. The method was applied as follows: the full scan range was set as 60 to 900 m/z. The resolution for MS1 and ddMS2 was set as 70,000 and 17,500, respectively. The maximum injection time for MS1 and ddMS2 was 100 ms and 45 ms, respectively. The automatic gain control for MS1 and ddMS2 was set as 3e6 and 2e5, respectively. The isolation window was 1.6 m/z. The normalized collision energies were set as 10/17/25 V and 30/40/50 V. The full scan method was applied as follows: full scan range, 60–900 m/z; resolution, 140,000; the maximum injection time, 100 ms; and automatic gain control, 3e6 ions.

### Data processing

Thermo Compound Discover 2.1 was used to process the raw data, following untargeted metabolomic analysis [18]. Compounds were identified using the mzCloud database (ddMS2). A similarity search was performed on all compounds identified in the ddMS2 data, using mzCloud. Thermo Compound Discoverer data quantification and annotation were performed using the package ropls in R software (R version 3.6.1).

Signal intensities were corrected for signal drift and batch effects by fitting a locally quadratic (Loess) regression model to the median intensity of the pooled QC samples. The alpha parameter (indicating span), which controlled smoothing, was set as 2 to avoid overfitting. The median area of all pooled QC samples was the same after correction.

Metabolites with a coefficient of variation >25 % in the QC samples were then filtered out because of their unstable quantifiability. The filtered compound areas were calibrated using the median, log transformed, and Pareto-scaled (Eqs. 1, 2, and 3).

1 $$Ni,j=\frac{Xi,j}{median(Xi)} median(X)$$

2 $$T\textrm{i},j=\log \left( Ni,j\right)$$

3 $$Si,j=\frac{Ti,j- mean(Tj)}{\sqrt{sd(Tj)}}$$

An orthogonal partial least squares discriminant analysis (OPLS-DA) model and a significance analysis of microarrays (SAM) model were used to compare the abundances of each metabolite. A cluster heat map was obtained by calculating the Pearson distance and generated using hclust in R.

### Pathway enrichment and correlation network analyses

The online software MetaboAnalyst 5.0 (https://www.metaboanalyst.ca/) was used to perform pathway enrichment analysis and correlation network analysis with Fisher’s exact test, in which metabolic pathways with *P* < 0.05 were considered significantly altered. We used the debiased sparse partial correlation algorithm, based on the desparsified graphical lasso modeling procedure, to construct the metabolite correlation network. The nodes of the network are the input metabolites, and the edges represent the correlations.

### Correlation analysis between metabolites and clinical markers

We selected clinical indicators including white blood cells, red blood cells, neutrophils, platelet counts, hemoglobin, MTX plasma concentration, alanine transaminase (ALT), aspartate aminotransferase (AST), serum creatinine, and blood urea nitrogen (BUN). The Spearman correlation was used to analyze the correlation between metabolites and clinical markers. *P* value < 0.05 was considered statistically significant.

## Results

**Fig. 1:**
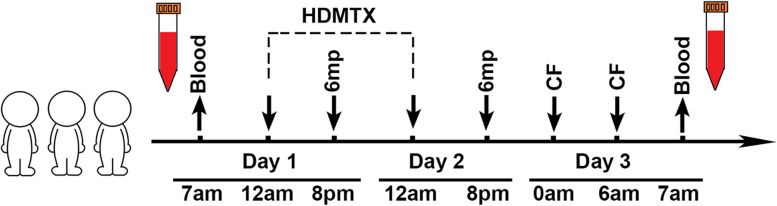
Schema of the consolidation medication, plasma collection timing, and untargeted metabolomic profiling of pediatric acute lymphoblastic leukemia (ALL) patients. Plasma was collected from each patient before and after consolidation therapy (on day 1 and day 3, respectively). HDMTX, high-dose methotrexate; 6-MP, 6-mercaptopurine; CF, calcium folinate

Figure 1 presents the study schema with plasma collection time points and a comprehensive untargeted metabolomics analysis. The baseline patient characteristics are shown in Table 1.

**Table 1:** Baseline patient characteristics and MTX- and 6-MP-associated gene polymorphisms

Characteristics	
Sex: F/M, *N*	15/12
Age rage in months, median (IQR)	13–152, 69 (44–132)
Risk group, *N* (%)^a^	Standard: 12 (44.4) Intermediate: 15 (55.6)
MRD46, *N* (%)^b^	< 0.01%: 23 (85.2) ≥ 0.01% < 0.1%: 3 (11.1) > 0.1%: 1 (3.7)

^a^Risk group was assigned based on age, leukocyte count, immunophenotype, central nervous system status, karyotype analysis, molecular status, and end-induction minimal residual disease (MRD) levels

^b^MRD46 (day 46) was determined on day 21 (±2) after cyclophosphamide, cytarabine, and 6-MP chemotherapy

### Metabolite detection

**Fig. 2:**
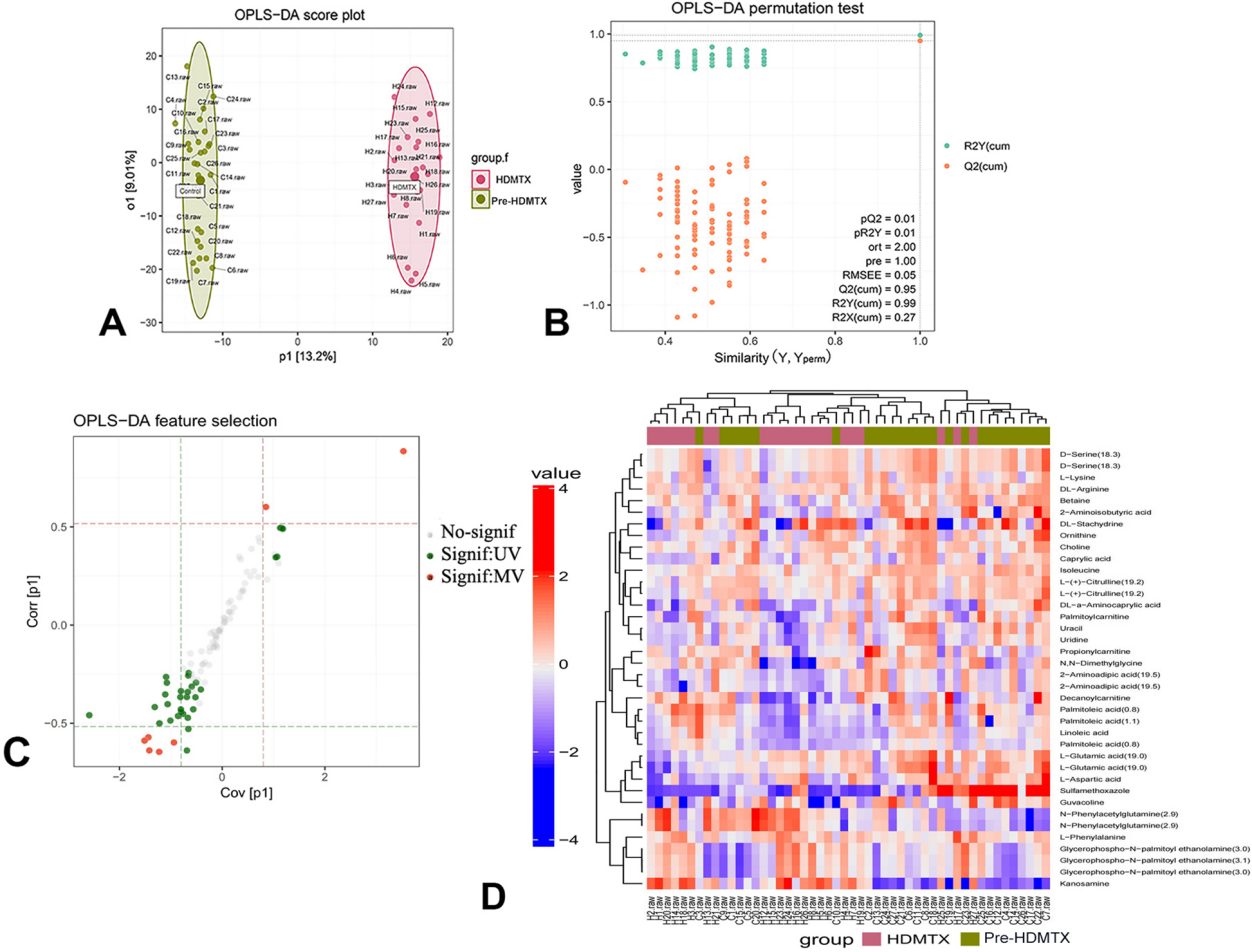
Untargeted metabolomic cluster analysis identified significant plasma metabolite responses to high-dose methotrexate (HDMTX) therapy in pediatric acute lymphoblastic leukemia (ALL) patients. **A** Score plot showing sample values without outliers. After being fitted to an OPLS-DA model, features in the raw dataset are collapsed into 1 predictive component and *i* ≥ 1 orthogonal components. Observations (samples) are represented by scores, which are linear combinations of the original variables with weights defined by the loadings. For each 2 components (generally predictive (p1) and orthogonal (o1)), a score plot can be generated by putting them in *X* and *Y* axes. **B** Permutation test results, revealing greater prediction performance. In the OPLS-DA model, the higher the Q2Y, the better the prediction performance. The *P* value is equal to the proportion of Q2Yperm above Q2Y. **C** S-plot of the predictive (p1) and orthogonal (o1) components, with significant feature points highlighted by OPLS-DA (MV) and SAM (UV). **D** Heat map of average metabolite signal intensity for the differential metabolites selected by SAM analysis

The 54 samples were randomized, and untargeted metabolomics was conducted using LC–MS/MS. After quality control, data filtering, and normalization (*n* = 27), 2826 metabolites were identified. An OPLS-DA model was established to screen the metabolites between the HDMTX group and pre-HDMTX group. Figure 2A displays an apparent difference between the two groups. In the OPLS-DA model, the permutation testing confirmed that the model was significant at the 0.01 level, which indicated that the model was reliable (Fig. 2B). The scatter plot (Fig. 2C) illustrated the significance and impact of the metabolic alterations following HDMTX therapy by OPLS-DA analysis and SAM analysis, and metabolites found using the SAM method were well overlapped with those identified in the OPLS-DA model. The highlighted feature points were considered significant compounds. Among them, SAM identified 38 metabolites, and the results of cluster analyses of differential metabolites between the two groups displayed differences in the expression of metabolites (Fig. 2D).

### Multivariate analysis of metabolomic alterations

**Fig. 3:**
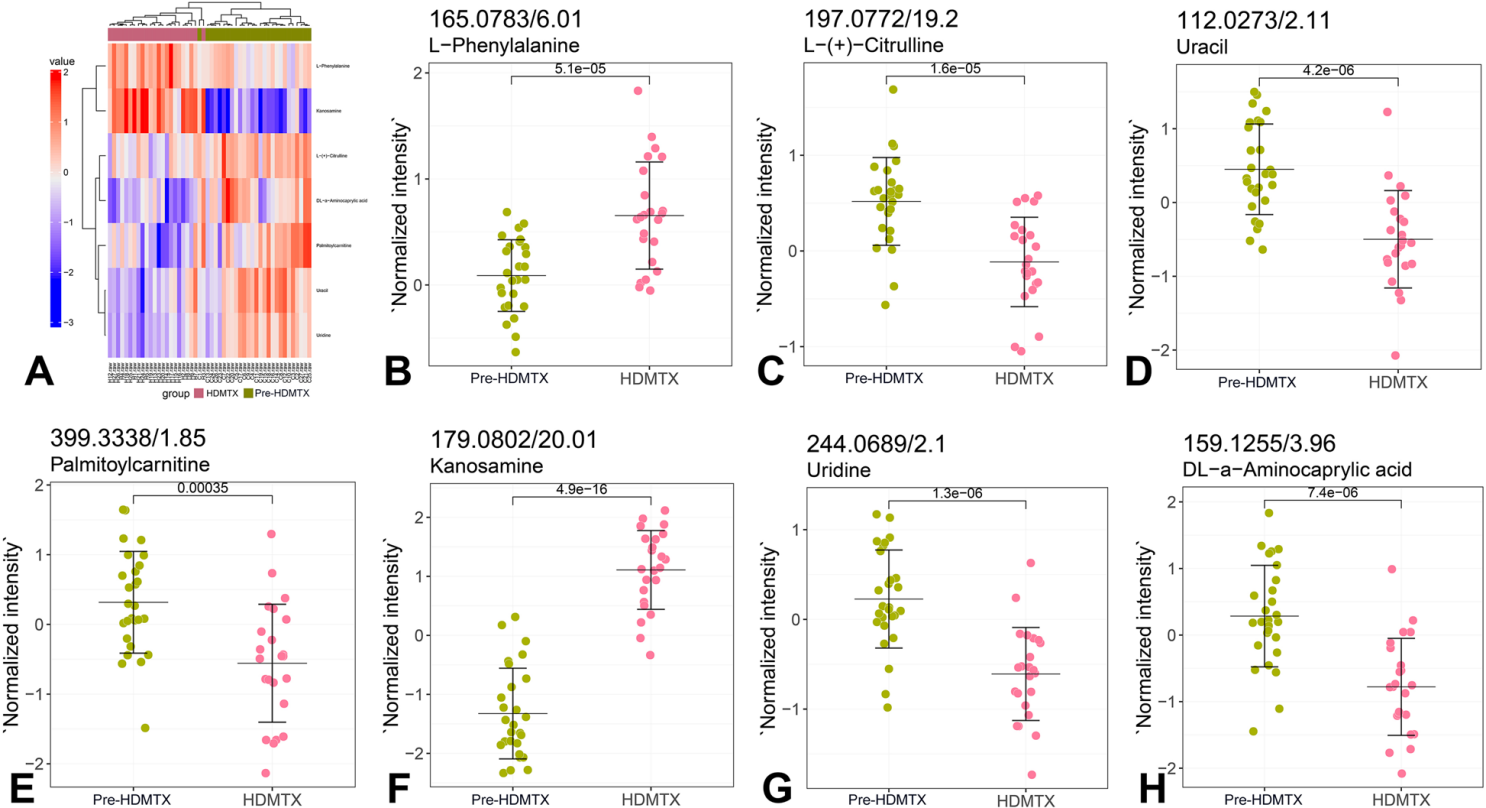
Metabolomic responses to high-dose methotrexate (HDMTX) therapy in pediatric acute lymphoblastic leukemia (ALL) patients. **A** Heat map showing the 7 differential metabolites identified. Blue-to-red color gradient: lower to higher metabolite levels (the color represents the average normalized intensity of each metabolite). **B**–**H** Scatter plots of changes in the significantly differential metabolites before (control) and after HDMTX treatment

The samples before and after HDMTX treatment were compared, and OPLS-DA analysis revealed 7 differential metabolites, which were illustrated via hierarchical clustering (Fig. 3A). Levels of l-phenylalanine and kanosamine were elevated following HDMTX treatment (Fig. 3B, F). In contrast, levels of l-(+)-citrulline (Fig. 3C), uracil (Fig. 3D), palmitoylcarnitine (Fig. 3E), uridine (Fig. 3G), and dl-a-aminocaprylic acid (Fig. 3H) were lower following HDMTX treatment.

### Metabolic pathway analysis

**Fig. 4:**
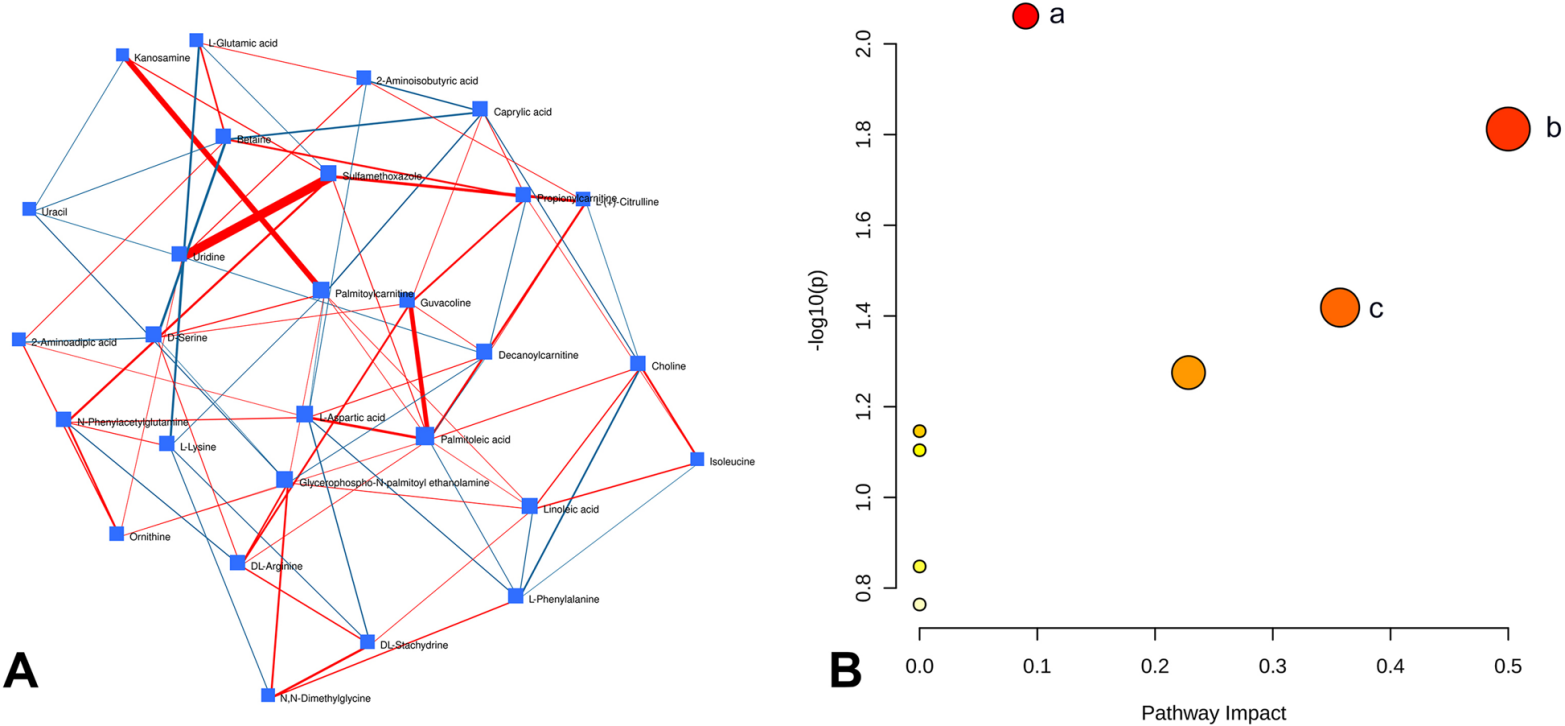
Metabolic pathways affected by high-dose methotrexate (HDMTX) in pediatric acute lymphoblastic leukemia (ALL) patients. **A** Regularized partial correlation network of the differential metabolites, following HDMTX therapy. Each node represents a compound, and each edge represents the strength of partial correlation between two compounds after conditioning on all other compounds in the dataset. The edge weights represent the partial correlation coefficients. **B** Bubble chart based on the KEGG pathway analysis of the significantly differential metabolites: (a) pyrimidine metabolism; (b) phenylalanine, tyrosine, and tryptophan biosynthesis; and (c) phenylalanine metabolism

We then examined the global pathways and regulatory relationships by constructing a regularized partial correlation network of the metabolites from OPLS-DA and SAM analysis to investigate the relationship between the 7 differential metabolites and other altered metabolites, which might find important compounds related to these differential 7 metabolites (Fig. 4A). Among them, uridine had the highest interaction density with sulfamethoxazole, and palmitoylcarnitine had a higher interaction density with kanosamine. L-Phenylalanine was negatively correlated with N,N-dimethylglycine and negatively correlated with choline. L-(+)-Citrulline also had a high interaction density with decanoylcarnitine (Fig. 4A).

**Fig. 5:**
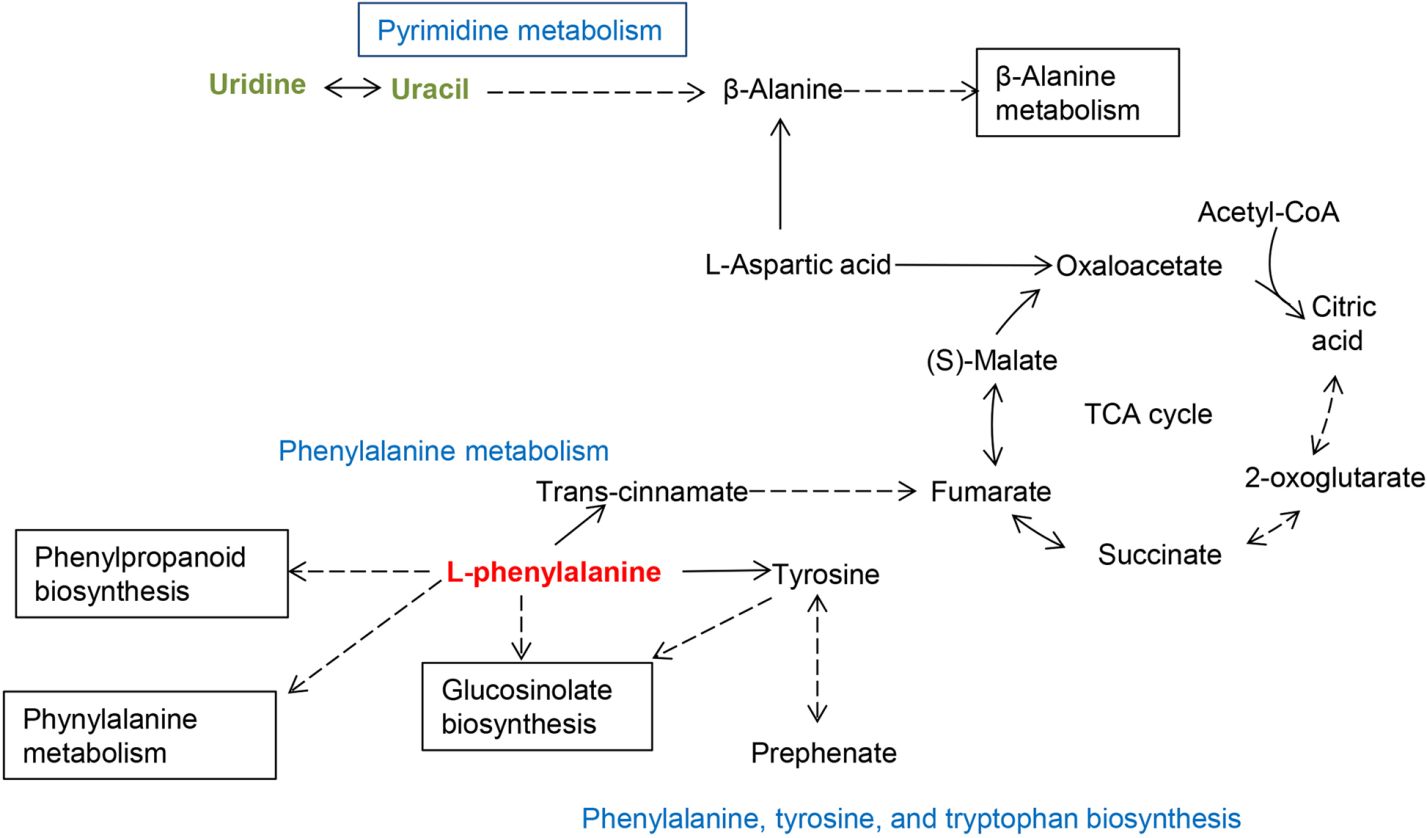
An integrated metabolic pathway after HDMTX treatment. Red metabolites represent upregulated metabolites, and green ones represent downregulated metabolites

Subsequently, 7 differential metabolites were subjected to KEGG for pathway enrichment analysis. Among the enriched KEGG pathways, MetaboAnalystR revealed that 3 primary metabolic pathways including pyrimidine metabolism; phenylalanine, tyrosine, and tryptophan biosynthesis; and phenylalanine metabolism were significantly affected by HDMTX treatment (*P* < 0.05; Fig. 4C and Table 2). Finally, a detailed metabolic pathway is shown in Fig. 5.

**Table 2:** Significantly affected pathways by HDMTX treatment

Pathways	*P*	−Log(*p*)	FDR	Impact
Pyrimidine metabolism	0.0087	2.0600	0.65	0.09
Phenylalanine, tyrosine, and tryptophan biosynthesis	0.0154	1.8100	0.65	0.50
Phenylalanine metabolism	0.0382	1.4200	1.00	0.36
Arginine biosynthesis	0.0531	1.2800	1.00	0.23
Pantothenate and CoA biosynthesis	0.0714	1.1500	1.00	0.00
Beta-alanine metabolism	0.0787	1.1000	1.00	0.00
Fatty acid degradation	0.1420	0.8480	1.00	0.00
Aminoacyl-tRNA biosynthesis	0.1720	0.7640	1.00	0.00

Metabolic pathways with *P* < 0.05 were considered significantly altered

### Correlations between differential metabolites and clinical indicators

**Fig. 6:**
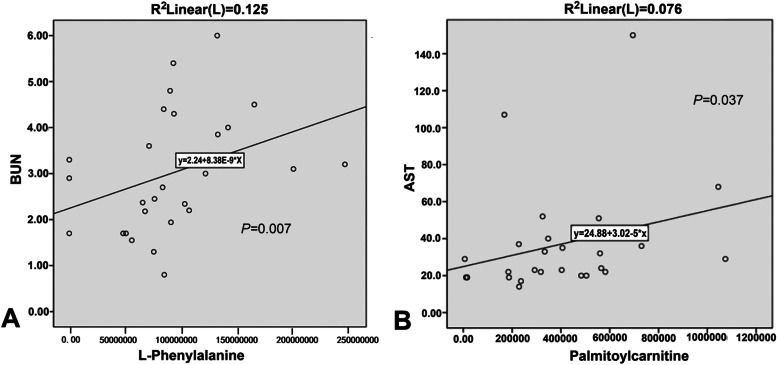
Correlations between metabolites and clinical markers, following high-dose methotrexate (HDMTX) treatment in pediatric acute lymphoblastic leukemia (ALL) patients. **A** L-Phenylalanine is correlated strongly with serum urea nitrogen. **B** Palmitoylcarnitine is correlated strongly with aspartate aminotransferase

We investigated the correlations between the levels of the 7 differential metabolites and clinical indicators, including white blood cells, red blood cells, neutrophils, platelet counts, hemoglobin, MTX plasma concentration, ALT, AST, serum creatinine, and BUN. The collection time points of all clinical indicators are the same as the sample collection time points. L-Phenylalanine and BUN levels were significantly correlated with each other (*P*  = 0 .007; Fig. 6A), like palmitoylcarnitine and AST (*P*  = 0 .037; Fig. 6B).

## Discussion

We aimed to elucidate the metabolic changes after HDMTX treatment, an important ALL chemotherapeutic, via an integrated LC–MS detection. Seven significantly altered metabolites were identified. Pathway analysis showed that HDMTX treatment mainly affected the pyrimidine metabolism; phenylalanine, tyrosine, and tryptophan biosynthesis; and phenylalanine metabolism pathways.

Metabolomic changes, reflecting genomic, transcriptomic, and proteomic variability, occur continuously [19]. Further, metabolic states are altered in diseases such as cancer and diseases of the liver, kidney, cardiovascular system, and nervous system [20, 21]. Metabolomics has been used to screen for potential diagnostic biomarkers at initial diagnosis and during early induction therapy in ALL. Schraw et al. [22] used LC–MS to profile end-of-induction-therapy plasma, marrow, and cerebrospinal fluid from children with B-ALL and find strong correlations between the biomarkers of clinically relevant phenotypes. In the study, we identified metabolite changes under HD-MTX therapy, described the metabolic signaling pathways involved, and expected to identify predictive metabolic markers for clinical side effects.

L-Phenylalanine is one of the 20 proteinogenic amino acids [23]. In humans, it is an essential amino acid and a precursor of tyrosine. At sufficiently high levels, phenylalanine acts as a neurotoxin [24] and metabotoxin [25]. Peng et al. [26] hypothesized that higher baseline phenylalanine levels indicate a greater risk of CNS leukemia. In all enrolled patients, there were no encephalatrophy, epilepsy, and clinical features of other encephalopathy when the central nervous system was evaluated. It has been reported that an L-phenylalanine polymer, Metabolic Reprogramming Immunosurveillance Activation Nanomedicine (MRIAN), can degrade into L-phenylalanine, which inhibits PKM2 activity and reduces ROS levels in myeloid-derived suppressor cells in T-cell acute lymphoblastic leukemia. These reactions lead to the disturbance of immunosuppressive function and are increased in the differentiation toward normal myeloid cells [27]. Song and colleagues demonstrated that there were 33 significantly altered metabolites between ALL patients with and without central nervous system involvement (CNSI), and the CNSI evaluation score was used to predict the risk of CNSI based on three independent risk factors (8-hydroxyguanosine, L-phenylalanine, and hypoxanthine), which could predict the diagnosis of ALL with CNSI [28]. Our finding revealed that L-phenylalanine was significantly elevated following HDMTX treatment and was enriched in phenylalanine, tyrosine, and tryptophan biosynthesis and phenylalanine metabolism. Besides, it was found that L-phenylalanine was positively correlated with BUN. The results might facilitate further investigation of whether renal dysfunction is associated with higher levels of L-phenylalanine in pediatric ALL.

Previous study has suggested that specific changes in polyamine, purine, and pyrimidine metabolism have been observed in patients with NPM1 mutations, which are a potential marker associated with favorable prognosis [29]. Moreover, pyrimidine metabolism is a network that can sense and modulate the amounts of deoxynucleotide, while resistance to decitabine and 5-azacytidine originates from adaptive responses of the pyrimidine metabolism network in myeloid malignancies [30]. The significant increases in levels of uracil and uridine in this study implied the disrupted pyrimidine metabolism after HDMTX treatment, which might provide a novel target for therapeutic response and prognosis prediction for ALL patients. Interestingly, it was also found that pyrimidine metabolism; phenylalanine, tyrosine, and tryptophan biosynthesis; and phenylalanine metabolism pathways were indirectly associated with the tricarboxylic acid (TCA) cycle. The TCA cycle is a key energy metabolic pathway, and the abnormal TCA cycle is implicated in cancer initiation. TCA cycle intermediates affect the processes of cancer development and progression via regulating cellular activities, such as metabolism and signaling [31]. Regulatory mechanisms of the TCA cycle and these 3 pathways remain to be further investigated.

Serum levels of DL-a-aminocaprylic acid, uracil, uridine, L-(+)-citrulline, and palmitoylcarnitine were significantly reduced following HDMTX treatment. The adverse reactions of HDMTX treatment included acute hepatic function damage where aspartate aminotransferase (AST) and alanine transaminase were mainly used as clinical indexes, acute renal dysfunction where BUN and creatinine were mainly used as clinical test indexes, and myelosuppression where white blood cells, red blood cells, and platelets were decreased. Palmitoylcarnitine levels were positively correlated with those of AST, which binds to mitochondria. Mitochondrial collapse during cellular necrosis dramatically increases serum AST [32]. DL-a-Aminocaprylic acid, a secondary metabolite that is metabolically non-essential, may serve as a defense or signaling molecule [33]. Uracil serves as an allosteric regulator and a coenzyme for many important biochemical reactions, helping to synthesize many enzymes necessary for cell function, by binding with riboses and phosphates [34]. Uridine, synthesized from uracil, participates in galactose glycolysis. Palmitoylcarnitine, a long-chain acyl fatty acid ester of carnitine, facilitates long-chain fatty acid transfer from the cytoplasm into mitochondria during fatty acid oxidation [34]. Generally, acylcarnitines transport acyl groups, organic acids, and fatty acids from the cytoplasm to mitochondria to be broken down to produce energy.

Given the clinical complexity of ALL and the limitations associated with logistic regression, these findings remain to be further verified. Studies with a larger sample size are required to detect significantly affected metabolic pathways. The plasma metabolome reflects extracellular metabolic changes in multiple organ systems, representing a potential limitation of our study. Independent-cohort validation is required to confirm these findings.

## Conclusion

Plasma metabolomic profiling provides a snapshot of metabolic changes [35], which are a good choice for the investigation on diseases and responses to treatment [36]. We identified 3 metabolic pathways that were significantly altered by HDMTX. Among the differential metabolites identified, L-phenylalanine, uridine, and uracil were distinctly enriched in pyrimidine metabolism; phenylalanine, tyrosine, and tryptophan biosynthesis; and phenylalanine metabolism after HDMTX therapy. Notably, HDMTX therapy affected L-phenylalanine, which was significantly correlated with BUN, and palmitoylcarnitine, which was significantly correlated with AST. Our findings indicated potential treatment mechanisms and biomarkers for HDMTX in pediatric ALL, thereby potentially improving therapeutic strategies for ALL.

## Supplementary Information

**Additional file 1: Supplementary Table 1.** The generic HPLC gradient.

## Data Availability

The datasets analyzed in this study can be found in the MetaboLights data repository (accession MTBLS4075).
