# Supplementary material for: Metabolic profiling reveals metabolic features of consolidation therapy in pediatric acute lymphoblastic leukemia
Source: Cancer Metab. 2023 Jan 23;11:2. doi: 10.1186/s40170-023-00302-6 (PMC9869545; doi:10.1186/s40170-023-00302-6)
Supplement: Supplementary file 1 — Additional file 1: Supplementary Table 1. The generic HPLC gradient. [file 40170_2023_302_MOESM1_ESM.docx]

**Supplementary Table 1:** The generic HPLC gradient

| Time | A | B |
| --- | --- | --- |
| 0.0 min | 10 % | 90 % |
| 1.0 min | 10 % | 90 % |
| 11.0 min | 13 % | 87 % |
| 140. min | 20 % | 80 % |
| 16.5 min | 30 % | 70 % |
| 18.5 min | 50 % | 50 % |
| 20.5min | 80 % | 20 % |
| 25.0min | 80 % | 20 % |
| 25.1 min | 10 % | 90 % |
| 34.0 min | 10 % | 90 % |
